# Supplementary figures and images for: The reciprocal regulation between mitochondrial-associated membranes and Notch signaling in skeletal muscle atrophy
Source: eLife. 2023 Dec 15;12:RP89381. doi: 10.7554/eLife.89381 (PMC10723794; doi:10.7554/eLife.89381)

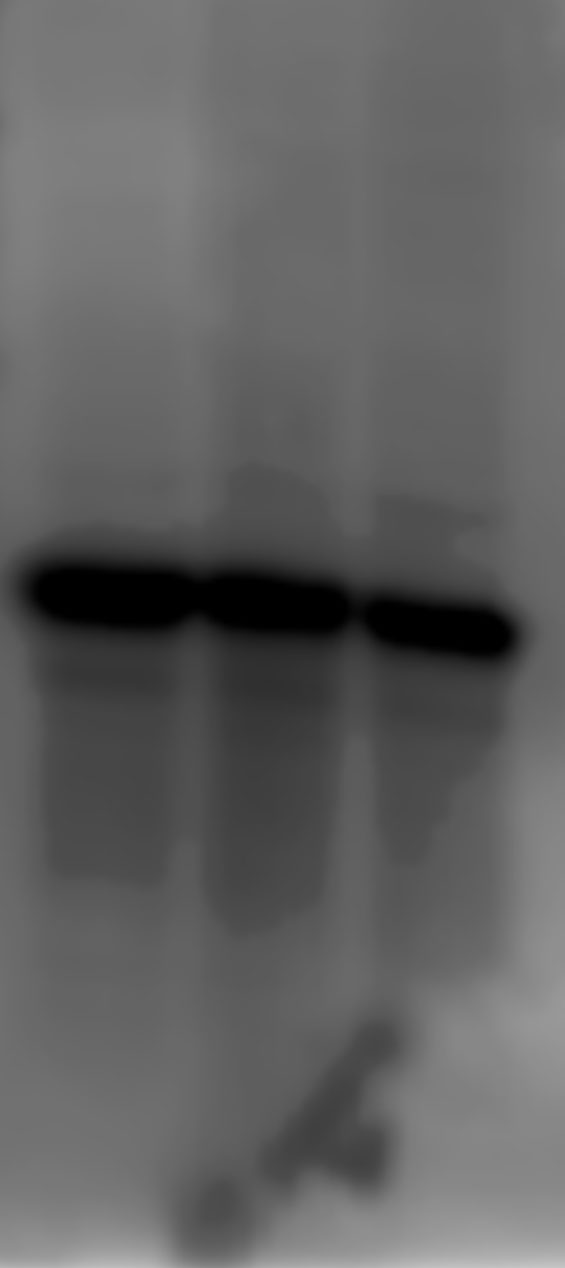

Supplement: Figure 1—source data 1. [file elife-89381-fig1-data1.zip › Fig1D/Fig1D_anti-GAPDH.jpg]

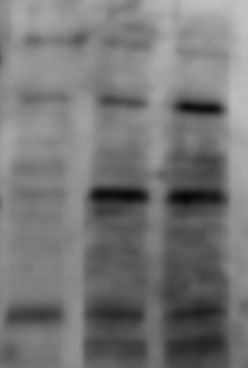

Supplement: Figure 1—source data 1. [file elife-89381-fig1-data1.zip › Fig1D/Fig1D_anti-TRIM63.jpg]

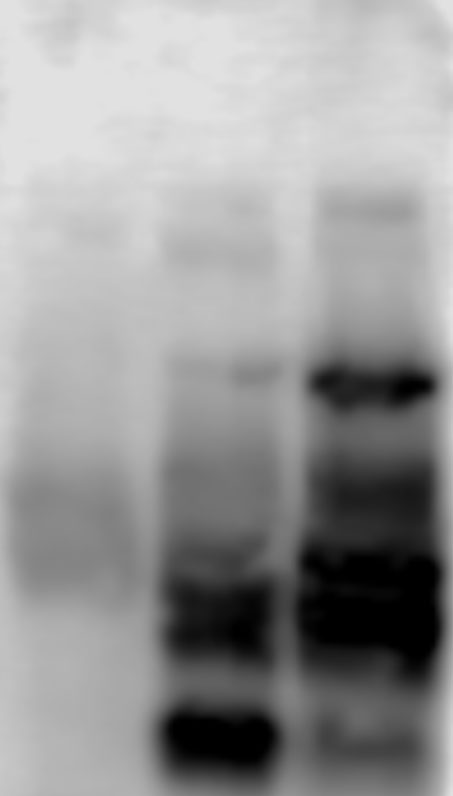

Supplement: Figure 1—source data 1. [file elife-89381-fig1-data1.zip › Fig1D/Fig1D_anti-FBXO32.jpg]

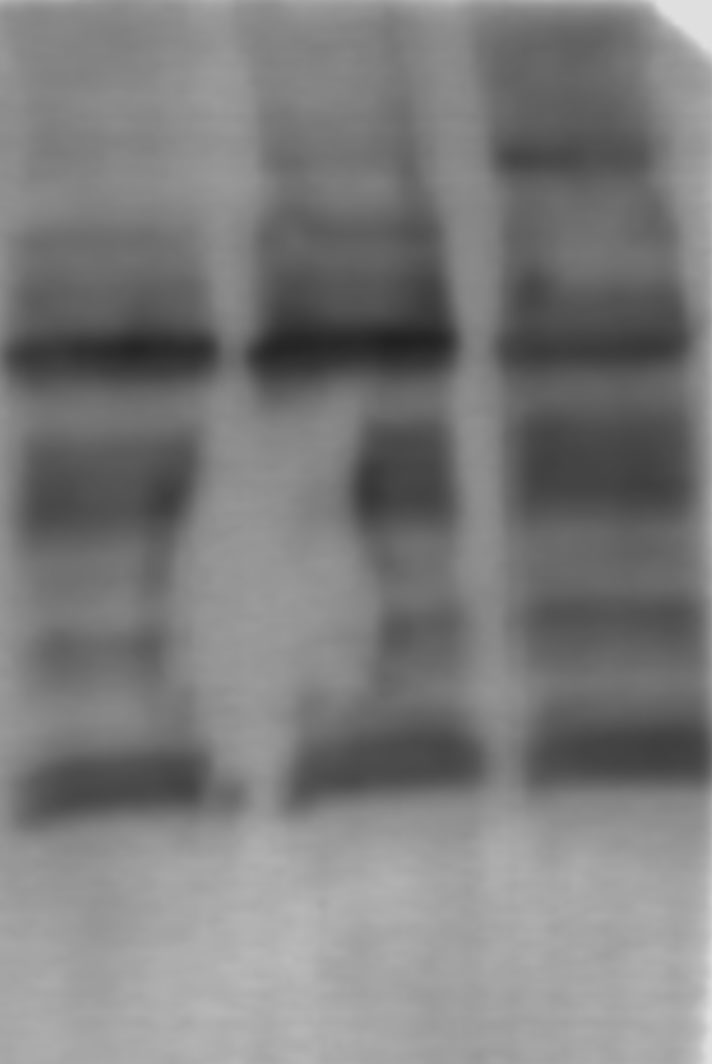

Supplement: Figure 1—figure supplement 3—source data 1. [file elife-89381-fig1-figsupp3-data1.zip › Fig1-s3/Fig1-S3_anti-AKT.jpg]

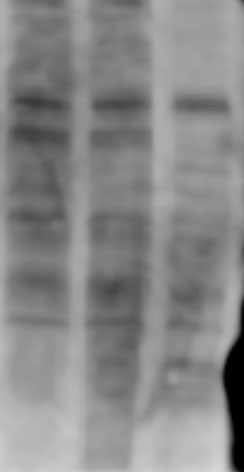

Supplement: Figure 1—figure supplement 3—source data 1. [file elife-89381-fig1-figsupp3-data1.zip › Fig1-s3/Fig1-S3_anti-pAKT.jpg]

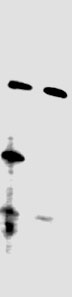

Supplement: Figure 5—source data 1. [file elife-89381-fig5-data1.zip › Fig5d/Fig5d_anti-histone H3.jpg]

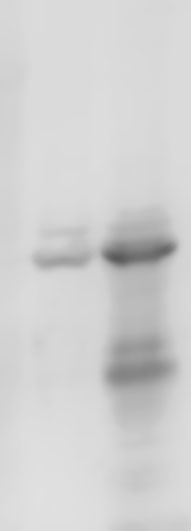

Supplement: Figure 5—source data 1. [file elife-89381-fig5-data1.zip › Fig5d/Fig5d_anti-NICD.jpg]
